# Controllable Polarization of Lasing Emission From a Polymer Microfiber Laser

**DOI:** 10.1038/s41598-019-53437-2

**Published:** 2019-11-19

**Authors:** Van Duong Ta, Rui Chen, Handong Sun

**Affiliations:** 1 Department of Optical Devices, Le Quy Don Technical University, Hanoi, 100000 Vietnam; 2 Division of Physics and Applied Physics, School of Physical and Mathematical Sciences, Nanyang Technological University, Singapore, 637371 Singapore; 3 Department of Electrical and Electronic Engineering, Southern University of Science and Technology, Shenzhen, Guangdong 518055 P.R. China; 4 Centre for Disruptive Photonic Technologies (CDPT), Nanyang Technological University, Singapore, 637371 Singapore; 5 MajuLab, CNRS-UCA-SU-NUS-NTU International Joint Research Unit, Singapore, 637371 Singapore

**Keywords:** Microresonators, Polymers

## Abstract

Microlasers with controllable polarization of output emission are vital for on-chip optical communications, optical sensors and optical switches. In this work, we report a high quality (Q) factor, low-threshold polymer microfiber laser and the possibility of achieving laser emission with a desired polarization. The microfiber is fabricated by direct drawing from a dye-doped polymer solution and it can generate whispering gallery mode (WGM) lasing under optical pulse excitation. When the microfiber is pumped from the side with pumping direction perpendicular to the microfiber’s axis, the polarization direction of the output laser is found to be the same as that of the pump laser. Lasing emission with either transverse electric (TE) or transverse magnetic (TM) modes can be obtained and these two polarization states can be switched over by tuning the pumping laser. Furthermore, emission with both TE and TM modes can also be observed by changing the orientation of the microfiber relatively to pumping direction. Our finding provides an effective approach for achieving microlasers that have high Q lasing modes with anticipated polarization.

## Introduction

Microlasers are potential for on-chip optical communications, medical imaging and biosensing^1^. Recently, organic microlasers, particularly polymer microlasers, are becoming increasingly competitive to conventional semiconductor microlasers due to their low cost, easy processing and mechanical flexibility^2,3^. Owing to the flexibility, polymers can be manipulated to different laser geometries such as thin membranes^4^, microspheres^5^, hemispheres^6^, microdisks^7^, microbubbles^8^ and micro-/nanofibers^9^. Among those geometries, polymer micro-/nanofibers are attractive because of their simple fabrication and wide range of applications in ultrafast photonics^10,11^, light modulation devices^12^, electronics and energy generation^13^, and optical solitons^14^.

Polymer fibers can be easily fabricated by common electrospinning approach^15^ or directly drawing from a polymer solution^16^. Polymer micro-/nanofibers are highly flexible that allows them to be assembled in a variety of closed-loop structures for random and network lasers^17,18^. Microlasers can be also realized on a single polymer microfiber. It has been demonstrated that a piece of dye-doped electrospun microfiber could support laser oscillation based on a Fabry-Perot (FP) cavity formed by their highly reflective end facets^19^. Portable and compact distributed feedback (DFB) lasers can also be achieved by directly imprinting on a single nanofiber^20^. Interestingly, polymer microfibers usually have a round cross-section, which makes them appropriate for high quality (Q) factor, low-threshold whispering gallery mode (WGM) microlasers^21^.

It is well-known that the polarization property of lasing emission is an essential characteristic of a WGM laser. Polarized light is vital to liquid crystal display (LCD) and optical divides such as optical modulators, optoisolators. Even though optically pumped polymer microfiber lasers have been reported^19–22^, the effect of pumping polarization and direction on the polarization property of output emission has been rarely studied. In this work, we demonstrate a high Q factor, low-threshold polymer microfiber laser and the ability to control the polarization of output laser.

## Results

### Surface chacterization of polymer fibers

**Figure 1:**
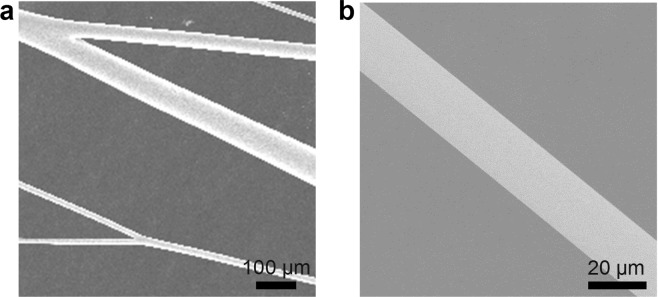
(**a**) SEM image of directly drawing polymer microfibers and (**b**) high magnification SEM image of a typical microfiber.

Figure 1a shows the SEM image of dye-doped polymer microfibers fabricated by directly drawing from a dye-doped polymer solution. Microfibers with various diameters ranging from about 10 to 100 µm could be obtained. As shown in Fig. 1b, fabricated polymer microfibers display cylindrical shape with a smooth outer surface, which is vital for the efficient optical confinement.

### Optical setup for studying the microfiber

**Figure 2:**
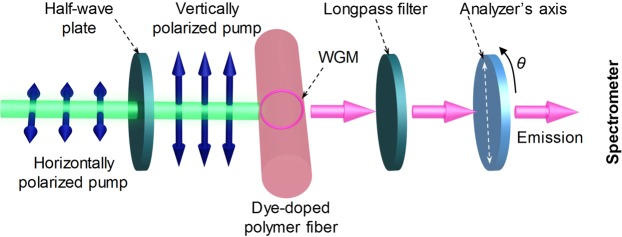
Illustration of the optical setup used for the realization of a polymer microfiber laser. The blue arrows denote the orientation of the electric field of the pump laser. *θ* represents the rotation angle of the analyzer. *θ* = 0° when the analyzer’s axis is parallel to the microfiber axis.

Emission from a WGM laser can be either transverse electric (TE) or transverse magnetic (TM) with distinct lasing wavelengths^23^. In a cylindrical fiber laser, TM modes have electric field parallel to the fiber axis while the electric field of TE modes is oscillating in a radial direction to the fiber centre^24^. As a result, TE and TM modes can be determined using a micro-photoluminescence (μ-PL) setup combined with a polarizer (as shown in Fig. 2). Initially, the polarizer’s axis is fixed to parallel to the fiber axis. Then, the polarizer is rotated clockwise under angle *θ*. As a result, lasing modes are TM (TE) if the output intensity is maximum at *θ* = 0°, 180° (90°) and minimum at 90° (0°, 180°).

### WGM lasing from the polymer fiber

**Figure 3:**
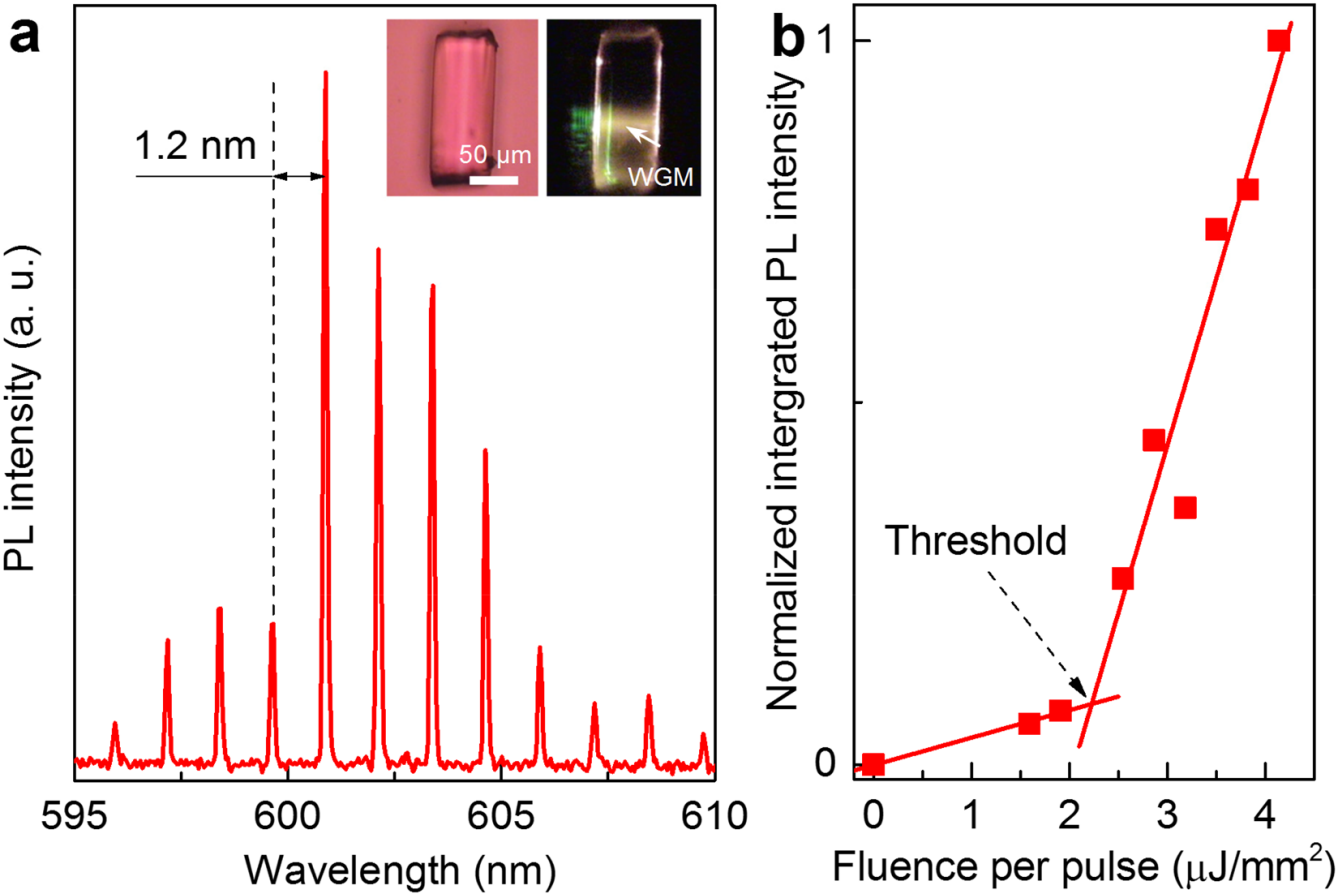
(**a**) A typical output lasing spectrum from the microfiber. The inset shows optical and PL microscope images of the studied microfiber, respectively. (**b**) Normalized output PL intensity from the polymer microfiber as a function of pump pulse fluence, exhibiting a laser threshold.

A piece of polymer microfiber was placed on top of a distributed Bragg reflector (DBR) whose has a high reflectivity (99%) at around 600 nm^25^. The DBR substrate is necessary as it prevents the optical leakage of emission from the microfiber to the substrate. Lasing emission was observed from the microfiber by optical pumping under room temperature and at ambient conditions. A typical lasing spectrum is shown in Fig. 3a, exhibiting a clear free-spectral range (FSR) of 1.2 nm. For a WGM cavity, the FSR can be estimated as *λ*^2^/π*nD*, where *λ* is resonant wavelengths; *n* and *D* are refractive index and diameter of the microfiber, respectively. Assuming, *λ* = 600 nm, *n* = 1.46 and *D* = 65 µm, calculated FSR is 1.2 nm, which is consistent with the experimental measurement. The result confirms WGM is the mechanism for laser generation in the microfiber. The spectral linewidth (δ*λ*) of the lasing mode is ~0.1 nm, corresponding to a Q factor of lasing mode, calculated by Q = *λ*/δ*λ*, is around 6 × 10^3^. Figure 3b plots the integrated output PL intensity from the microfiber as a function of pumping fluence, indicating a distinct lasing threshold of ~2.2 µJ/mm^2^ (~7 µJ per pulse).

### Polarization property of WGM lasing from the polymer fiber

**Figure 4:**
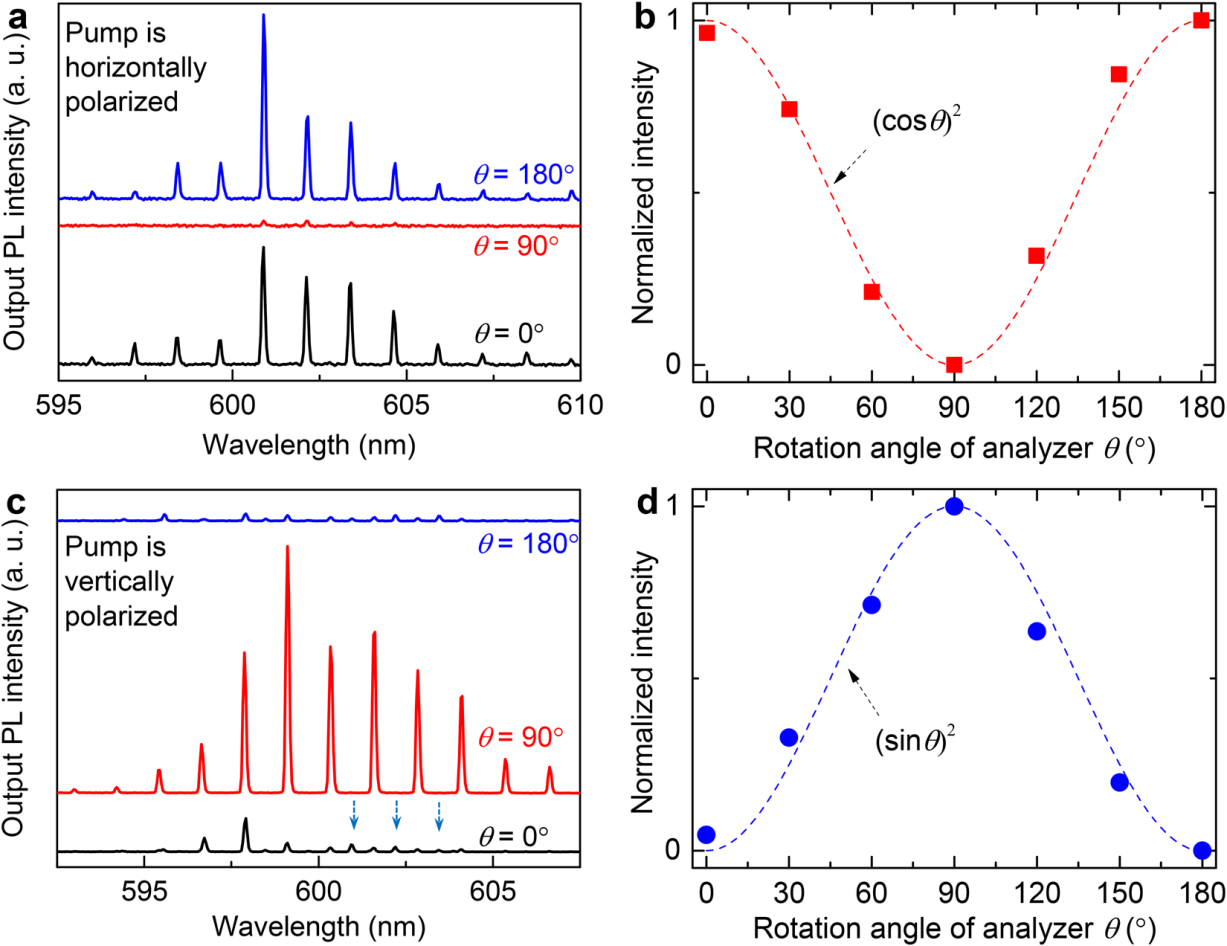
(**a,c**) Output lasing spectra from the microfiber for various *θ* of 0°, 90°, 180°, under laser excitation with horizontal and vertical polarization, respectively. The arrows in (**c**) highlight low-intensity TM modes. (**b,d**) Normalized output lasing intensity from the microfiber as a function of *θ* under optical excitation with horizontal and vertical polarization, respectively.

Analysis of output emission from the microfiber (placed vertically) reveals that lasing peaks have the same polarization as pumping laser. When the pump was horizontally polarized, the output lasing peaks were primarily of TM characteristics. As shown in Fig. 4a, lasing modes with similar intensity can be clearly observed at *θ* = 0°, 180° and highly suppressed at *θ* = 90°. It is found that the lasing intensity can be well-fitted by (cos*θ*)^2^ function or the Malus’s law (Fig. 4b), which confirms the TM characteristics of the lasing modes. In contrast, when the pump was vertically polarized, the output lasing peaks are maximum at *θ* = 90° and minimum at *θ* = 0°, 180° (Fig. 4c). TM modes were also appeared (represented as arrows) but with much lower intensity. The observation of TM modes suggests that the TM modes are easier to generate in comparison with TE modes, which may due to the anisotropic configuration of the fiber. The integrated intensity of lasing modes as a function of *θ* is now well-fitted with (sin*θ*)^2^ function (Fig. 4d), hence lasing peaks were TE polarized modes. A similar result was previously observed in fiber lasers based on evanescent-wave-coupled gain using a glass capillary^26^. It is expected that fiber lasers whose have similar lasing configuration and using dye molecules as the active medium would exhibit the same polarization property. The result demonstrates that the polarization of lasing emission from the polymer fiber laser can be directly controlled by the polarization of the optical pumping beam, which might be promising for applications in optical switches^27^.

It is well-known that the pump laser determines the polarized states of the excited dye molecules and consequently polarization property of output laser^26^. Also, the orientation of dye molecules during the pumping process also affect the polarization of output laser. Herein, as the microfiber is solid, dye molecules are fixed inside the polymer matrix during the pumping and subsequent lasing. As a result, the pump laser directly determines the polarization property of the microfiber laser. This case is not the same for liquid droplet lasers. It has been reported that the molecular reorientation time of Rhodamine dye is around 250 ps that leads to the observation of both TE and TM modes lasing^23^.

**Figure 5:**
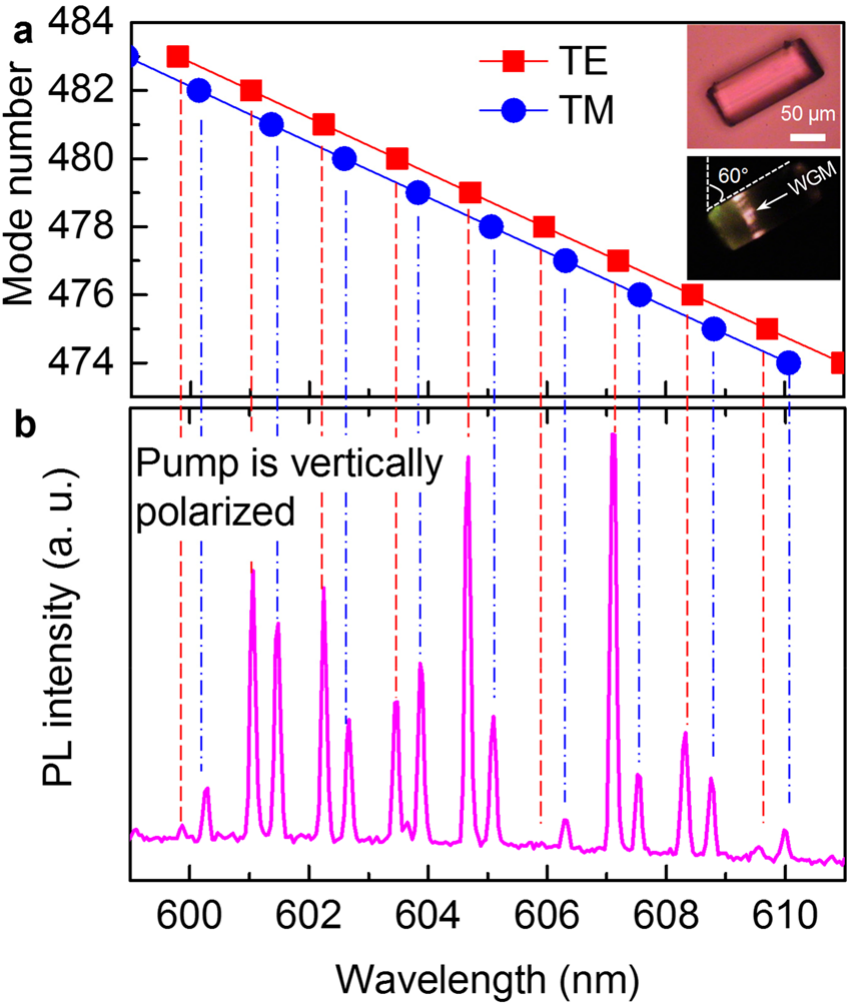
(**a**) Calculated mode numbers (*m*) for TE and TM modes that are well-fitted with (**b**) the corresponding output lasing spectrum from the microfiber. The inset shows optical and PL microscope images of the studied microfiber that is located at an inclined angle of 60° to the vertical direction.

From the observation of TE and TM modes, it is expected that if the polarization of the pump beam is adjusted between the horizontal and vertical direction, the emission would have both TE and TM modes. We tested this hypothesis by rotating the microfiber instead of rotating the polarization state of the pump laser. To make sure both TE and TM modes are visible, the fiber was rotated 30° (the smallest tuning step) anticlockwise to the horizontal direction. By doing so, we obtained two sets of lasing modes as shown in Fig. 5. Analysis of lasing wavelengths discloses that one lasing envelope was TE polarized while the other was TM polarized. The lasing modes of the two sets are well fitted by explicit asymptotic formulas^28^:

1 $${({\lambda }_{m}^{q})}^{-1}=\frac{1}{\pi D{n}_{1}}[v+{2}^{-1/3}\,{A}_{q}{v}^{1/3}-\frac{P}{\sqrt{{n}_{r}^{2}-1}}+\frac{{2}^{-2/3}{A}_{q}^{2}{v}^{-1/3}}{10/3}-\frac{P({n}_{r}^{2}-\frac{2}{3}{P}^{2}){A}_{q}}{{2}^{1/3}{({n}_{r}^{2}-1)}^{3/2}{v}^{2/3}}]$$

where, *D* is the diameter of the circular microcavity; *ν* = *m* + 0.5, *m* is the mode number; *n*_r_ = *n*_1_/*n*_2_ with *n*_1_ and *n*_2_ are refractive index of the microfiber and outside medium, respectively; *A*_q_ is the roots of the Airy function, where *q* is the mode order; *A*_q_ = 2.338 for fundamental mode *q* = 1. *P* is a coefficient related to the polarization property. *P* = *n*_r_ for TE modes and *P* = 1/*n*_r_ for TM modes. Assuming *D* = 65.965 µm and *n* = 1.46, resonant wavelengths of mode number *m* = 474–483 (Fig. 5a) are matched well with emission wavelengths (Fig. 5b), which confirms the TE and TM characteristic of the lasing modes.

The incline of the microfiber should explain the appearance of both TE and TM modes in the emission spectrum. If imaging the electric field of the pumping laser is a vector, then this vector is equal to a sum of two distinct components. The first component parallels to the microfiber axis would generate TM modes while the other perpendiculars to the microfiber axis would generate TE modes. The amplitude of the parallel component is half of the vertical one because the inclined angle of the microfiber is 60° to the vertical direction. As a result, it is expected that the TE modes would have a higher intensity compared with the TM modes. This expectation was observed and shown in Fig. 5. The result indicates that the relative intensity of TE and TM modes may be controlled by simply changing the orientation of the microfiber.

## Discussion

We have demonstrated a polymer microfiber laser with a lasing threshold of 2 µJ/mm^2^ and a Q factor of 6 × 10^3^. When the microfiber was pumped from the side, the pump laser determines the polarization property of the microfiber laser. Lasing emission with TM (TE) modes was obtained when pump laser has electric field parallel (perpendicular) to the microfiber axis. Both TE and TM modes were observed when the microfiber placed at an inclined angle to the pumping direction. The result suggests that pumping polarization and direction have a significant effect on the polarization property of the polymer microfiber laser. Therefore, these two factors may be used to control the output polarization of microfiber lasers. Our finding may be potential for applications in all-polymer-fiber optical switches, optical modulators and optical sensors.

In addition, it has been demonstrated that FP lasing would be possible when the edges of an electrospun microfiber are cleaved^29^. Smooth and flat end faces increase reflectivity from the edges of the microfiber, providing significant axial feedback for laser realization. As a result, it is expected that FP lasing from our microfiber is also possible if the edges of the microfiber are cleaved. That means two different lasing mechanisms, WGM and FP, with distinct properties can be generated from a single structure. This kind of laser would be interesting for future investigation as it offers additional flexibility such as laser wavelength and direction of emission for laser-based applications.

## Methods

### Fabrication of the polymer fiber

The polymer microfiber was fabricated by directly drawing from a dye-doped solution^16^. The polymer solution was prepared by subsequently dissolving Polymethylmethacrylate (PMMA), Rhodamine 6G (R6G) and Araldite 506 epoxy resin in dichloromethane (DCM) (all chemical are from Sigma-Aldrich). The concentration of PMMA, RhB and epoxy resin in DCM are around 11, 0.06 and 22 wt %, respectively. As a result, the dry ratio of PMMA, R6G, the epoxy resin in fabricated microfibers are 33.3, 0.2, and 66.5 wt%, respectively.

### Optical measurements

A polymer microfiber was investigated by using a micro-photoluminescence (μ-PL) setup. Pumping source was a frequency-doubled, Q-switched Nd:YAG laser (wavelength: 532 nm, pulse width: 1 ns, frequency: 60 Hz). Intrinsically, the pumping laser is horizontally polarized. However, its polarization can be rotated 90°, becoming vertically polarized, by using a half-wave plate. A green laser beam was guided at an angle ∼45° to the normal of the substrate and focused on a 1 mm-diameter spot to excite the microfiber. PL emission from excited the microfiber was collected from the top by an objective (50×, NA = 0.42) and subsequently delivered to a camera for PL image and a spectrometer for spectral recording. A longpass filter and a polarizer were inserted in front of the spectrometer for blocking the pump light and for analyzing the polarization of output emission, respectively.
